# Sacrospinous Ligament Fixation After Failed Sacrocolpopexy: A Case Series

**DOI:** 10.1002/ccr3.71305

**Published:** 2025-10-20

**Authors:** Yaman Degirmenci, Gilbert Georg Klamminger, Andrea Appel, Matthias Alexa

**Affiliations:** ^1^ Department of Obstetrics and Gynecology University Medical Center of the Johannes Gutenberg University Mainz Mainz Germany

**Keywords:** apical pelvic organ prolapse, case report, sacrocolpopexy, sacrospinous ligament fixation

## Abstract

Recurrent apical prolapse after failed sacrocolpopexy poses a surgical challenge, with limited evidence on surgical treatments. This case series evaluates the feasibility and 1‐year outcomes of sacrospinous ligament fixation (SSLF) of the residual cervix in this setting. Three postmenopausal women with symptomatic recurrent apical prolapse underwent SSLF after prior laparoscopic sacrocolpopexy. At 3‐ and 12‐month follow‐up, all patients showed sustained apical support (POP‐Q C −8), with significant improvement in symptoms and quality of life (ICIQ‐VS scores). Mild, asymptomatic anterior compartment descent was noted in two cases. SSLF of the cervix appears to be a safe, effective, and minimally invasive alternative to redo sacrocolpopexy for selected patients with recurrent apical prolapse.

## Graphical abstract

Sacrospinous ligament fixation of the residual cervix provides safe and durable apical support after failed sacrocolpopexy, offering a minimally invasive alternative to redo sacrocolpopexy.

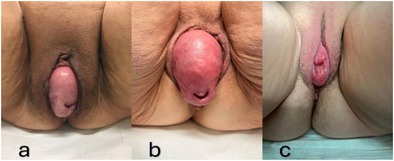

**Summary**

- Recurrent apical prolapse after failed sacrocolpopexy is challenging, with limited guidance. While redo sacrocolpopexy is standard, less invasive vaginal options are underexplored.
- This case series presents 1‐year outcomes of sacrospinous ligament fixation (SSLF) after failed laparoscopic sacrocervicopexy, suggesting SSLF as a safe, effective alternative for selected patients.

## 1 Introduction

Although the exact incidence of pelvic organ prolapse (POP) remains challenging to determine, epidemiological studies suggest that up to one‐third of adult women experience symptoms related to this condition [1]. While mild POP may spontaneously regress, moderate prolapse often progresses and may ultimately require surgical intervention [2]. Literature estimates suggest that the lifetime risk of undergoing POP surgery is between 10% and 20%, with reoperation rates nearing 30% [1, 3].

However, the anterior compartment is the most frequently affected location in pelvic organ prolapse (POP), typically manifesting as a cystocele. Its underlying pathophysiology is often associated with an apical defect, typically presenting as a concurrent clinical finding [4]. When surgical correction of apical prolapse is indicated, sacrocolpopexy (SCP) has been shown to provide lower rates of prolapse awareness, objective recurrence, and reoperation rates compared to various vaginal procedures [5]. SCP, widely regarded as the gold standard for apical prolapse repair, demonstrates success rates ranging from 74% to 98% in published literature [6]. The overall reoperation rate following laparoscopic SCP is approximately 15%, accounting for both urinary incontinence and mesh‐related complications [7]. The reoperation rate for pelvic organ prolapse after laparoscopic SCP is, on the other hand, only about 5%, with recurrences most commonly involving the anterior and posterior compartments [8]. Recurrent apical prolapse following SCP is even less common, occurring in fewer than 1% of cases, where reoperation may be necessary [9].

Although the rates of reoperation due to failure or complications following SCP have been extensively discussed and well documented in the literature, only a few studies have addressed the surgical management of recurrent apical prolapse, explicitly focusing on the so‐called “redo sacrocolpopexy,” albeit with limited information on the choice of surgery, the explicit surgical technique for reoperation, or intraoperative findings [6, 8, 10, 11, 12, 13, 14, 15]. To date, only two studies have described a transvaginal approach for treating recurrent apical prolapse after failed SCP. These techniques include uterosacral ligament suspension, transvaginal utilization of the existing sacrocolpopexy graft, and placement of a new transvaginal prosthesis, all demonstrating acceptable anatomical outcomes [16, 17]. Overall, redo sacrocolpopexy remains a technically demanding procedure for treating recurrent POP after failed apical repair. Despite reports of high success rates, the current evidence base remains limited regarding apical recurrent prolapse after failed SCP, highlighting the need for further well‐designed studies to guide clinical decision‐making.

Since its introduction, sacrospinous ligament fixation (SSLF) has become a well‐established vaginal approach for apical prolapse, with satisfactory success rates. When long‐term anatomical support and preservation of sexual function are prioritized, sacrocolpopexy is often the preferred technique. However, SSLF may offer advantages over SCP, including lower mesh‐related risk, reduced operative time, decreased cost, and fewer wound‐related complications [18]. However, in the context of uterus‐preserving surgery, sacrospinous hysteropexy and laparoscopic sacrohysteropexy appear equally effective for the long‐term management of uterine prolapse, with comparable rates of both objective and subjective recurrence rates [19, 20]. To our knowledge, no published reports specifically evaluate SSLF for treating recurrent prolapse after failed SCP, including long‐term follow‐up data.

This report aims to assess whether sacrospinous fixation of the residual cervix represents a viable treatment option for recurrent apical pelvic organ prolapse following laparoscopic supracervical hysterectomy with sacrocervicopexy. Written informed consent was obtained from all patients for this case report.

## 2 Case History/Examination

**FIGURE 1:**
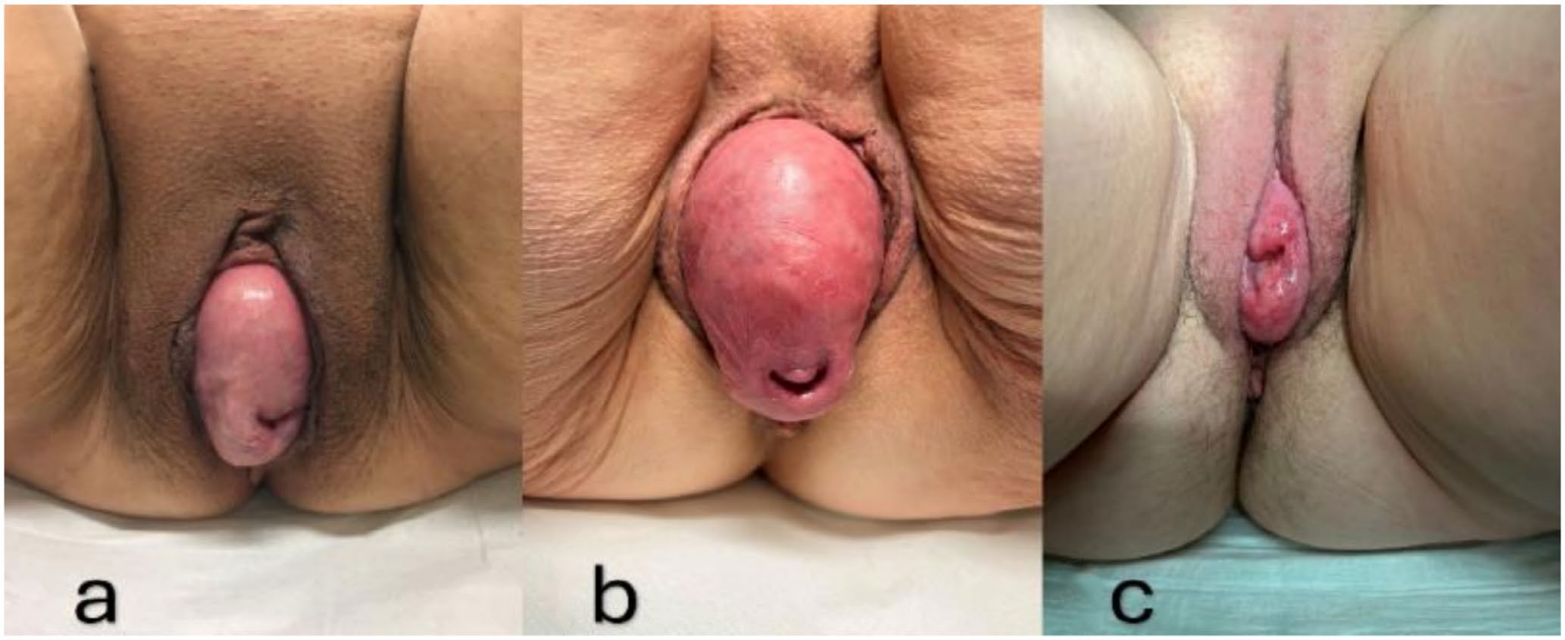
Clinical presentation of prolapse at the time of initial presentation (a: Case 1; b: Case 2; c: Case 3).

This case series describes sacrospinous ligament fixation of the cervix as a treatment for recurrent apical prolapse following failed SCP. Three non‐sexually active postmenopausal women were referred to our center between October 2022 and April 2023 with symptoms of vaginal bulging and urinary frequency due to recurrent apical prolapse following laparoscopic supracervical hysterectomy with SCP for apical POP. Clinical examination revealed recurrent prolapse, with a third‐degree prolapse of the cervix and bladder according to the POP‐Q classification in Case 1 (Ba +6, C +7) (Figure 1a), and Case 2 (Ba +6, C +7) (Figure 1b). In Case 3, there was merely a third‐degree prolapse of the cervix (regarding POP‐Q scores: Ba −2, C +2) (Figure 1c). According to the patients' histories, recurrence was observed in Case 1 at 6 months, in Case 2 at 6 weeks, and in Case 3 at 4 months after the initial operation. Vaginal symptoms and quality of life (QOL) scores, as assessed by the ICIQ‐VS (vaginal symptoms questionnaire), were 42/10 (Case 1), 26/8 (Case 2), and 15/10 (Case 3).

Investigations and treatment: Clinical investigations included routine transvaginal ultrasonography to assess the pelvic organs and transperineal ultrasonography to evaluate urethral dynamics and visualize the bladder, rectum, central compartment, and, when applicable, the mesh position. As these findings had no impact on surgical management, further diagnostics such as pelvic MRI were not performed. After discussing various treatment options, such as redo SCP, sacrospinous ligament fixation of the cervix was selected as the least invasive surgical approach. In Cases 1 and 2, an anterior colporrhaphy with a one‐layer closure using a delayed absorbable suture was initially performed to correct the accompanying cystocele. A posterior colpotomy below the cervix followed this to expose the sacrospinous ligament via a posterior approach. Sacrospinous ligament fixation of the cervix was then performed using the classical technique, with two non‐absorbable sutures placed to ensure adequate apical support. Follow‐up evaluations were scheduled for 3 and 12 months postoperatively to monitor outcomes.

### 2.1 Outcome and Follow‐Up

**FIGURE 2:**
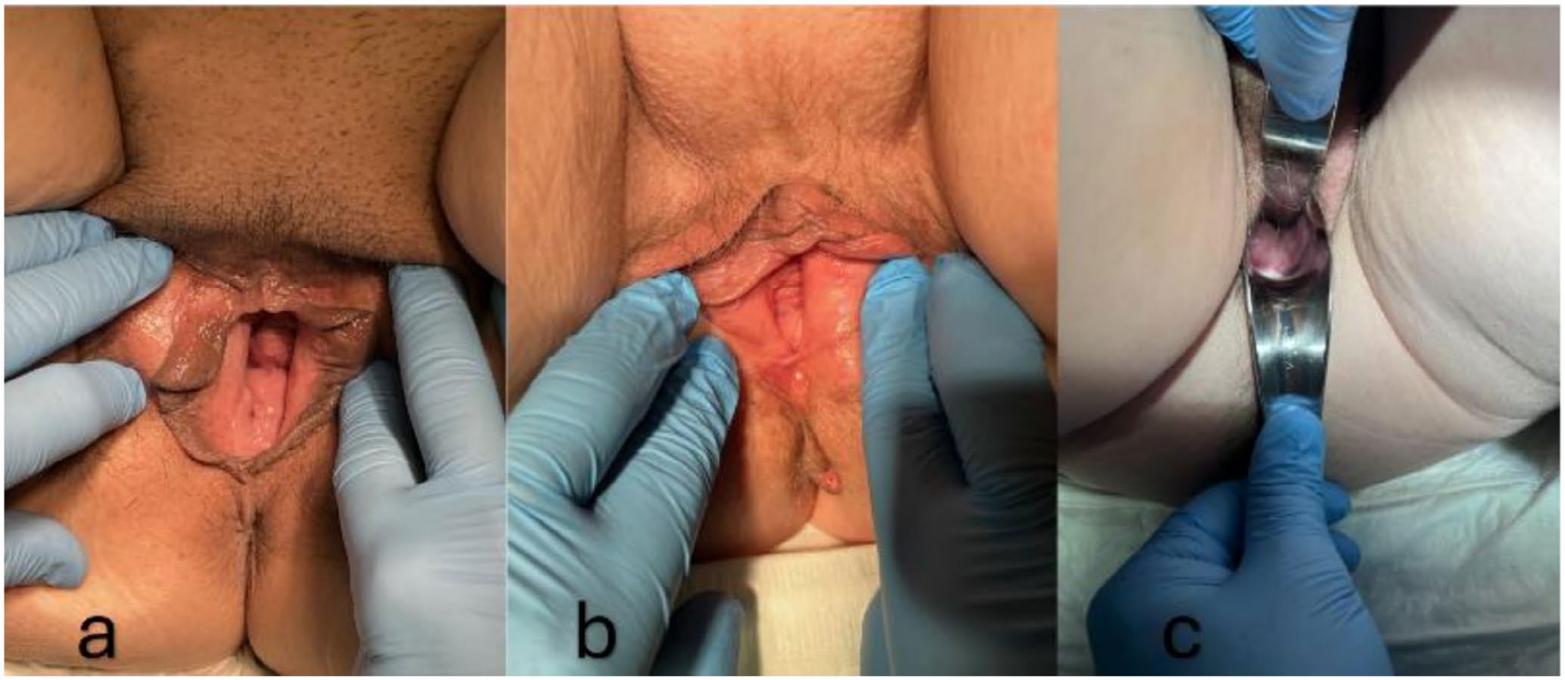
Clinical presentation of prolapse at the time of first follow‐up (a: Case 1; b: Case 2; c: Case 3).

**FIGURE 3:**
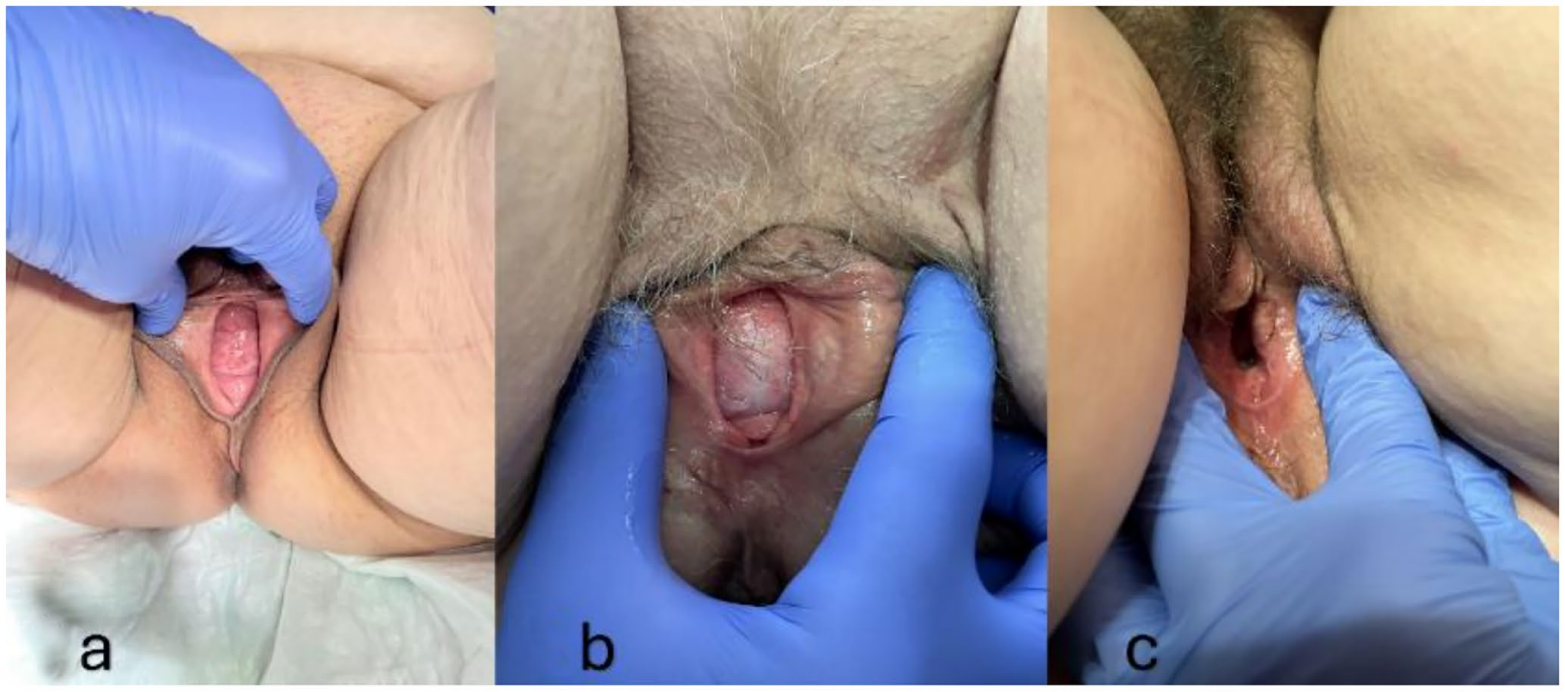
Clinical presentation of prolapse at the time of second follow‐up (a: Case 1; b: Case 2; c: Case 3).

During the first follow‐up (approximately 3 months after surgery), the clinical examination revealed proper apical fixation and sufficient stabilization of the anterior vaginal wall in all cases, according to the POP‐Q classification: Case 1 (Ba −3, C −8) (Figure 2a), Case 2 (Ba −2, C −8) (Figure 2b), and Case 3 (Ba −3, C −8) (Figure 2c). Vaginal symptom scores and quality of life (QOL) scores significantly improved in all cases, as measured by the ICIQ‐VS: 10/3 (Case 1), 2/0 (Case 2), and 7/1 (Case 3). The second follow‐up was set approximately 12 months after the initial surgery. Hereby, the clinical examination showed maintained apical fixation in all cases and solely moderate and asymptomatic prolapse of the anterior vaginal wall in Cases 1 and 2, resulting in the following POP‐Q classifications: Case 1 (Ba −1, C −8) (Figure 3a), Case 2 (Ba −1, C −8) (Figure 3b), and Case 3 (Ba −3, C −8) (Figure 3c). Case 1 developed increasing symptoms of anal incontinence, which had been relatively masked before the surgery. Vaginal symptom scores and quality of life (QOL) scores remained improved in Cases 2 and 3, while Case 1 reported a decreased quality of life due to anal incontinence, as measured by the ICIQ‐VS: 28/10 (Case 1), 6/0 (Case 2), and 3/1 (Case 3).

## 3 Discussion

Based on our results, SSLF of the remnant cervix provides optimal apical support even at 1 year postoperatively, serving as a valuable rescue option after failed sacrocolpopexy. The presence of the cervical ring and ligamentous structures, along with the refixation of the cervix via a vaginal approach, may enhance success rates compared with purely vaginal fixation, particularly in cases of vaginal vault prolapse. Supporting evidence in the literature indicates that sacrospinous hysteropexy, as an organ‐preserving procedure compared with vaginal hysterectomy, demonstrates overall better anatomical outcomes and excellent patient‐reported outcomes, with no prolapse symptoms at the 12‐month follow‐up, even when anatomical recurrence of the anterior vaginal wall was taken into account [21, 22]. However, while the anatomical success rates for simultaneous cystocele repair remain moderate, the subjective outcomes are satisfactory, consistent with the known results of anterior colporrhaphy reported in the literature [23]. The altered angle following sacrospinous fixation and the resulting enlargement of the space in the anterior compartment may contribute to the anatomical recurrence of cystocele in our cohort [24].

Pelvic floor reconstruction should aim to provide adequate support with minimal perioperative morbidity. The safety and time efficiency of vaginal surgery must also be carefully considered in the management of pelvic organ prolapse (POP) recurrence after laparoscopic procedures. In this regard, another advantage of SSLF is its reduced duration and compatibility with spinal anesthesia, making it particularly suitable for elderly individuals and patients with comorbid conditions. Moreover, the less complex learning process compared with SCP facilitates its wider dissemination and accessibility [18]. Sacrospinous ligament fixation can also be performed in a fast‐track setting in a cohort with a high rate of previous prolapse surgery, yielding comparable subjective and objective outcomes with traditional approaches and a low complication rate. In contrast, redo sacrocolpopexy may be associated with higher conversion rates and, although not statistically significant, with increased rates of reintervention for prolapse compared with primary sacrocolpopexy [8, 25]. A less invasive approach, consistent with the nature of vaginal prolapse surgery, offers the potential to substantially improve quality of life and patient satisfaction. A strong evidence‐based statement regarding the treatment of recurrence after failed sacrocolpopexy cannot be made based on the available literature. However, a comparable case study evaluating vaginal surgery after failed sacrocolpopexy, comparing the utilization of the pre‐existing mesh with bilateral traditional uterosacral ligament fixation, avoiding re‐attachment to the failed mesh, demonstrates superior outcomes with the traditional ligament fixation, further highlighting the value of traditional vaginal surgery in managing recurrence after failed sacrocolpopexy [16]. In conclusion, SSLF of the cervix appears to be a feasible and effective option for the management of apical prolapse following SCP failure.

## Author Contributions

**Yaman Degirmenci:** conceptualization, data curation, writing – original draft, writing – review and editing. **Gilbert Georg Klamminger:** supervision, writing – original draft, writing – review and editing. **Andrea Appel:** supervision, writing – original draft, writing – review and editing. **Matthias Alexa:** supervision, data curation, writing – original draft, writing – review and editing.

## Ethics Statement

Ethical approval was not required for this case series.

## Consent

Written informed consent for publication was obtained from all patients included in this case series.

## Conflicts of Interest

The authors declare no conflicts of interest.

## Data Availability

All datasets are available within the manuscript. Additional information is available upon request from the corresponding author.

## References

[1] E. Lowenstein , B. Ottesen , and H. Gimbel , “Incidence and Lifetime Risk of Pelvic Organ Prolapse Surgery in Denmark From 1977 to 2009,” International Urogynecology Journal 26, no. 1 (2015): 49–55. 24842118 10.1007/s00192-014-2413-y

[2] V. L. Handa , E. Garrett , S. Hendrix , E. Gold , and J. Robbins , “Progression and Remission of Pelvic Organ Prolapse: A Longitudinal Study of Menopausal Women,” American Journal of Obstetrics and Gynecology 190, no. 1 (2004): 27–32. 14749630 10.1016/j.ajog.2003.07.017

[3] A. L. Olsen , V. J. Smith , J. O. Bergstrom , J. C. Colling , and A. L. Clark , “Epidemiology of Surgically Managed Pelvic Organ Prolapse and Urinary Incontinence,” Obstetrics and Gynecology 89, no. 4 (1997): 501–506. 9083302 10.1016/S0029-7844(97)00058-6

[4] G. M. Northington , C. O. Hudson , D. R. Karp , and S. A. Huber , “Concomitant Apical Suspensory Procedures in Women With Anterior Vaginal Wall Prolapse in the United States in 2011,” International Urogynecology Journal 27, no. 4 (2016): 613–619. 26630948 10.1007/s00192-015-2894-3

[5] C. Maher , E. Yeung , N. Haya , et al., “Surgery for Women With Apical Vaginal Prolapse,” Cochrane Database of Systematic Reviews 7, no. 7 (2023): CD012376. 37493538 10.1002/14651858.CD012376.pub2 PMC10370901

[6] E. Grinstein , O. Gluck , N. Veit‐Rubin , and B. Deval , “Laparoscopic Management of Pelvic Organ Prolapse Recurrence After Open Sacrocervicopexy,” International Urogynecology Journal 31, no. 9 (2020): 1965–1968. 32222793 10.1007/s00192-020-04283-8

[7] D. Vandendriessche , J. Sussfeld , G. Giraudet , J. P. Lucot , H. Behal , and M. Cosson , “Complications and Reoperations After Laparoscopic Sacrocolpopexy With a Mean Follow‐Up of 4 Years,” International Urogynecology Journal 28, no. 2 (2017): 231–239. 27549223 10.1007/s00192-016-3093-6

[8] E. Bauters , A. S. Page , L. Cattani , et al., “Safety and Medium‐Term Outcome of Redo Laparoscopic Sacrocolpopexy: A Matched Case‐Control Study,” International Urogynecology Journal 34, no. 11 (2023): 2799–2807. 37632537 10.1007/s00192-023-05631-0

[9] C. O. Hudson , G. M. Northington , R. H. Lyles , and D. R. Karp , “Outcomes of Robotic Sacrocolpopexy: A Systematic Review and Meta‐Analysis,” Female Pelvic Medicine & Reconstructive Surgery 20, no. 5 (2014): 252–260. 25181374 10.1097/SPV.0000000000000070 PMC4374352

[10] M. Malekzadeh , L. Ramirez‐Caban , and E. Hurtado , “Laparoscopic Excision and Re‐Attachment of Sacrocolpopexy Mesh,” International Urogynecology Journal 32, no. 12 (2021): 3301–3303. 34003310 10.1007/s00192-021-04818-7

[11] A. I. Goodwin , J. Torres , D. L. O'Shaughnessy , and P. S. Finamore , “A Robotic Approach to Management of Failed Sacrocolpopexy and Sacrocolpopexy Complications: A Case Series,” International Urogynecology Journal 33, no. 11 (2022): 3231–3236. 35267061 10.1007/s00192-022-05134-4

[12] E. Ruess , J. P. Roovers , and S. Jeffery , “Management of Recurrent Pelvic Organ Prolapse After Sacrocolpopexy. A Video Case Series,” International Urogynecology Journal 31, no. 7 (2020): 1483–1485. 31915843 10.1007/s00192-019-04222-2

[13] G. Panico , G. Campagna , L. Vacca , et al., “Redo Laparoscopic Sacrocolpopexy for POP Recurrence: Is It the Right Call?,” European Journal of Obstetrics, Gynecology, and Reproductive Biology 276 (2022): 63–68. 35809460 10.1016/j.ejogrb.2022.06.023

[14] A. M. Studer , I. Faehnle‐Schiegg , J. Frey , S. Aichner , C. Brambs , and C. Christmann‐Schmid , “Recurrent Pelvic Organ Prolapse After Sacrocolpopexy—A Surgical Challenge,” Journal of Clinical Medicine 13, no. 6 (2024): 1613. 38541839 10.3390/jcm13061613 PMC10970834

[15] U. Omosigho , M. F. R. Paraiso , and O. H. Chang , “Revision Sacrocolpopexy: Tips and Tricks for Optimal Outcomes,” International Urogynecology Journal 34, no. 3 (2023): 783–785. 36181549 10.1007/s00192-022-05370-8

[16] J. N. Bracken , D. H. Tran , T. J. Kuehl , W. Larsen , P. M. Yandell , and B. L. Shull , “A Novel Transvaginal Approach to Correct Recurrent Apical Prolapse After Failed Sacral Colpopexy: Case Series,” International Urogynecology Journal 23, no. 10 (2012): 1429–1433. 22527557 10.1007/s00192-012-1762-7

[17] T. S. Lo , R. Ibrahim , N. B. Karim , E. A. Nawawi , and M. C. Uy‐Patrimonio , “Trans‐Vaginal Mesh Surgery for Management of Recurrent Pelvic Organ Prolapse Following Abdominal Sacrocolpopexy,” Taiwanese Journal of Obstetrics & Gynecology 57, no. 2 (2018): 311–314. 29673679 10.1016/j.tjog.2018.02.023

[18] W. Zhang , W. C. Cheon , L. Zhang , X. Wang , Y. Wei , and C. Lyu , “Comparison of the Effectiveness of Sacrospinous Ligament Fixation and Sacrocolpopexy: A Meta‐Analysis,” International Urogynecology Journal 33, no. 1 (2022): 3–13. 34081163 10.1007/s00192-021-04823-w PMC8739324

[19] V. I. J. MN , A. M. van Oudheusden , J. Veen , et al., “Hysteropexy in the Treatment of Uterine Prolapse Stage 2 or Higher: Laparoscopic Sacrohysteropexy Versus Sacrospinous Hysteropexy—A Multicentre Randomised Controlled Trial (LAVA Trial),” BJOG: An International Journal of Obstetrics and Gynaecology 127, no. 10 (2020): 1284–1293. 32267624 10.1111/1471-0528.16242

[20] A. M. J. van Oudheusden , A. W. M. Coolen , H. Hoskam , J. Veen , and M. Y. Bongers , “Laparoscopic Sacrohysteropexy Versus Vaginal Sacrospinous Hysteropexy as Treatment for Uterine Descent: Comparison of Long‐Term Outcomes,” International Urogynecology Journal 34, no. 1 (2023): 211–223. 35482083 10.1007/s00192-022-05185-7 PMC9834108

[21] R. J. Detollenaere , J. den Boon , J. Stekelenburg , et al., “Sacrospinous Hysteropexy Versus Vaginal Hysterectomy With Suspension of the Uterosacral Ligaments in Women With Uterine Prolapse Stage 2 or Higher: Multicentre Randomised Non‐Inferiority Trial,” BMJ 351 (2015): h3717. 26206451 10.1136/bmj.h3717 PMC4512203

[22] S. F. M. Schulten , R. J. Detollenaere , J. Stekelenburg , J. IntHout , K. B. Kluivers , and H. W. F. van Eijndhoven , “Sacrospinous Hysteropexy Versus Vaginal Hysterectomy With Uterosacral Ligament Suspension in Women With Uterine Prolapse Stage 2 or Higher: Observational Follow‐Up of a Multicentre Randomised Trial,” BMJ (Clinical Research Ed.) 366 (2019): l5149. 10.1136/bmj.l5149 PMC6734519 31506252

[23] E. Valtersson , K. R. Husby , M. Elmelund , and N. Klarskov , “Evaluation of Suture Material Used in Anterior Colporrhaphy and the Risk of Recurrence,” International Urogynecology Journal 31, no. 10 (2020): 2011–2018. 32638062 10.1007/s00192-020-04415-0

[24] E. H. Sze , J. Meranus , N. Kohli , J. R. Miklos , and M. M. Karram , “Vaginal Configuration on MRI After Abdominal Sacrocolpopexy and Sacrospinous Ligament Suspension,” International Urogynecology Journal and Pelvic Floor Dysfunction 12, no. 6 (2001): 375–379. 11795640 10.1007/s001920170016

[25] S. Greisen , S. M. Axelsen , K. M. Bek , R. Guldberg , and M. Glavind‐Kristensen , “Fast Track Sacrospinous Ligament Fixation: Subjective and Objective Outcomes at 6 Months,” BMC Women's Health 21, no. 1 (2021): 154. 33863314 10.1186/s12905-021-01309-1 PMC8051023

